# Pristine and hydrated fluoroapatite (0001)

**DOI:** 10.1107/S2052520619010412

**Published:** 2019-09-10

**Authors:** Xavier Torrelles, Immad M. Nadeem, Anna Kupka, Adrián Crespo-Villanueva, Sandrina Meis, Hermann Gies, Oier Bikondoa

**Affiliations:** a Institute of Materials Science of Barcelona (ICMAB-CSIC), Campus UAB, Bellaterra, Barcelona 08193, Spain; b London Centre for Nanotechnology and Department of Chemistry, University College London, 20 Gordon Street, London WC1H OAJ, UK; c Harwell Science and Innovation Campus, Diamond Light Source Ltd, Didcot, Oxfordshire OX11 0DE, UK; d Faculty of Geosciences, Department of Geology, Mineralogy and Geophysics, Ruhr-Universität Bochum, Universitätsstrasse 150, Bochum 44780, Germany; e Department of Physics, University of Warwick, Gibbet Hill Road, Coventry CV4 7AL, UK; f XMaS, The UK-CRG Beamline, ESRF The European Synchrotron, CS40220, F-38043 Grenoble cedex 09, France

**Keywords:** fluoroapatite (0001), water interface, surface structure, mineralogy, surface X-ray diffraction

## Abstract

The surface structure of fluoro­apatite (0001) (FAp_0001_) under quasi-dry and humid conditions has been probed with surface X-ray diffraction (SXRD). Lateral and perpendicular atomic relaxations corresponding to the FAp_0001_ termination before and after H_2_O exposure and the location of the adsorbed water molecules have been determined from experimental analysis of the crystal truncation rod (CTR) intensities. The surface under dry conditions exhibits a bulk termination with relaxations in the outermost atomic layers. The hydrated surface is formed by a disordered partially occupied H_2_O layer containing one water molecule (33% surface coverage) adsorbed at each of the three surface Ca atoms, and is coupled with one OH group randomly bonded to each of the three topmost P atoms with a 33% surface coverage.

## Abstract 2

The surface structure of fluoro­apatite (0001) under dry and humid conditions has been probed with X-ray diffraction. The dry surface shows incomplete tetrahedra on the surface that are partially filled when the surface is hydrated.

## 1. Introduction   

Apatites represent a group of phosphate minerals that are very common in nature. Their generic chemical formula is Ca_5_(PO_4_)_3_ *X*, where *X* corresponds to a monovalent anion such as F^−^, (OH)^−^ *etc*. Structurally, apatites crystallize in the hexagonal space group *P*6_3_/*m* (No. 176) where the hexagonal axis is normal to a symmetry plane (Bragg *et al.*, 1965 ▸). The apatite structure is built of unconnected [PO_4_] tetrahedra, with Ca^2+^ cations occupying the space between the tetrahedra, and the *X* ^−^ anions compensating the extra charge of the Ca^2+^ cations and located along the *c* axis (Calderín *et al.*, 2003 ▸). Fluoro­apatite (calcium fluorophosphate, FAp) is a typical member of the apatite group with *X* = F. It contains 42 atoms per unit cell and has two formula units (*Z* = 2) per unit cell.

FAp is often found as an accessory mineral in igneous rocks, in the form of elongated prismatic needle-shaped crystal inclusions (Haldar & Tišljar, 2014 ▸). The substitution of small amounts (∼4 to 7 wt%) of PO_4_^3−^ by CO_3_^2−^ leads to carbon­ated FAp, which is an important sink in the marine phosphorus cycle (Ruttenberg & Berner, 1993 ▸). Apatites are also used as a source of phosphate for fertilizer production. Apatitic structures are also very relevant in biomineralization (Combes *et al.*, 2016 ▸). Calcium hy­droxy­apatite is found in bones, and human enamel and dentine are composed of hy­droxy­apatite (HAp, *X* = OH). FAp may also be found, in varying proportions, in human enamel that has been exposed to F^−^ ions, as FAp is chemically more stable than HAp. HAp and FAp are both used in regenerative medical techniques (Ben-Nissan, 2014 ▸). In some animal species (*e.g.* cartilaginous fish) instead of HAp enamel, the species have FAp enameloid, although the dentine is HAp (Lübke *et al.*, 2015 ▸). Teeth with FAp dentine have been found in the fossil record, but the presence of FAp in the dentine is due to diagenesis and exchange between the hydroxyl group and fluoride ions (Renzi *et al.*, 2016 ▸). The diagenetic transformation from HAp to FAp may depend on the microstructure of the original tissue (Lübke *et al.*, 2017 ▸).

The mineral–water interface plays a fundamental role in the functions of apatite both in geochemistry and in medicine and biomineralization. Previously, we investigated the surface structure of orthorhombic FAp (100) (FAp_100_) under dry and humid conditions [relative humidity (RH) ∼75%] using data from surface X-ray diffraction experiments (SXRD). The interface in a humid atmosphere shows a well organized and periodically ordered monolayer of water arranged laterally, with atomic relaxations of the mineral surface structure smaller than those of the dry surface (Pareek *et al.*, 2007 ▸). Here we extend this study to the hexagonal FAp (0001) (FAp_0001_) termination of a natural FAp single crystal under dry conditions (in an He-saturated environment) followed by exposure to a water-saturated environment at ambient temperatures.

## 2. Experimental and SXRD analysis details   

A natural FAp single crystal from Durango, Mexico, with a (0001) termination was rinsed with ultrapure water (resistivity 18.2 MΩ cm), sonicated in ethanol (Sigma–Aldrich, purity >99.8%) and lastly mounted within a small Kapton tent in which He gas (Air Liquide, purity 5 N) was flowing continuously during the dry measurements. Next, water vapour was introduced into the tent by bubbling helium through an ultrapure water (resistivity 18.2 MΩ cm) reservoir and an ultrapure-water-soaked paper tissue was placed in the tent. The SXRD experiments were carried out on beamline BM28 (XMaS) at the European Synchrotron Radiation Facility (ESRF). All measurements were performed in a vertical four-circle geometry at a constant incidence angle of 0.5° and photon energy of 15 keV (https://warwick.ac.uk/fac/cross_fac/xmas/). To follow surface geometry changes qualitatively, the intensity of the (−1, −1, 1.5) reflection was monitored. The index of this reflection, and all others hereafter, is expressed using Bravais–Miller indices (Hahn, 1996 ▸) and in terms of the reciprocal-lattice vectors *h*, *k* and *l*. These vectors are defined with reference to the real-space (1 × 1) unit cell of FAp_0001_ described by the lattice vectors **a**, **b** and **c** which are parallel to the 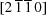, 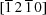 and [0001] directions, respectively. The magnitudes of these lattice vectors are *a* = *b* = 9.375 Å and *c* = 6.8870 Å and the angles are α = β = 90° and γ = 120° (Elliot, 1994 ▸).

A total of 581 and 704 reflections were measured under dry and humid conditions, respectively, which reduced to 535 [dry, 14 crystal truncation rods (CTRs) + reflectivity] and 620 (humid, 16 CTRs + reflectivity) non-equivalent reflections, respectively. The structure of the surface was determined adopting the usual approach: theoretical SXRD data are generated for a potential structure and then the model structure is iteratively refined to find the best fit between the experimental and theoretical structure factors. A modified version of the *ROD* software – a modification that allows the user to form blocks of molecules and rotate them around a common origin – was used for this task (Vlieg, 2000 ▸; Torrelles *et al.*, 2004 ▸). The goodness-of-fit was evaluated in terms of two commonly used parameters, χ^2^ (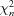) (Feidenhans’l, 1989 ▸) and the *R* factor (Stout & Jensen, 1968 ▸):
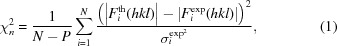


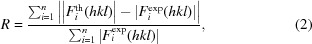
where *N* is the number of measured structure factors, *P* is the number of parameters optimized during the fitting procedure, and 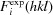 and 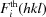 are the experimental and theoretically calculated structure factors, respectively. 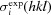 is the uncertainty associated with 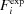 and was estimated by averaging equivalent reflections. 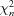 behaves such that a value of 1 indicates good agreement between the experimental and theoretically calculated structure factors. The quoted precision of each fitted parameter is determined by varying the parameter about its optimal values until 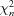 has increased by 1/(*N* − *P*) from its minimum value (Feidenhans’l, 1989 ▸).

**Figure 1:**
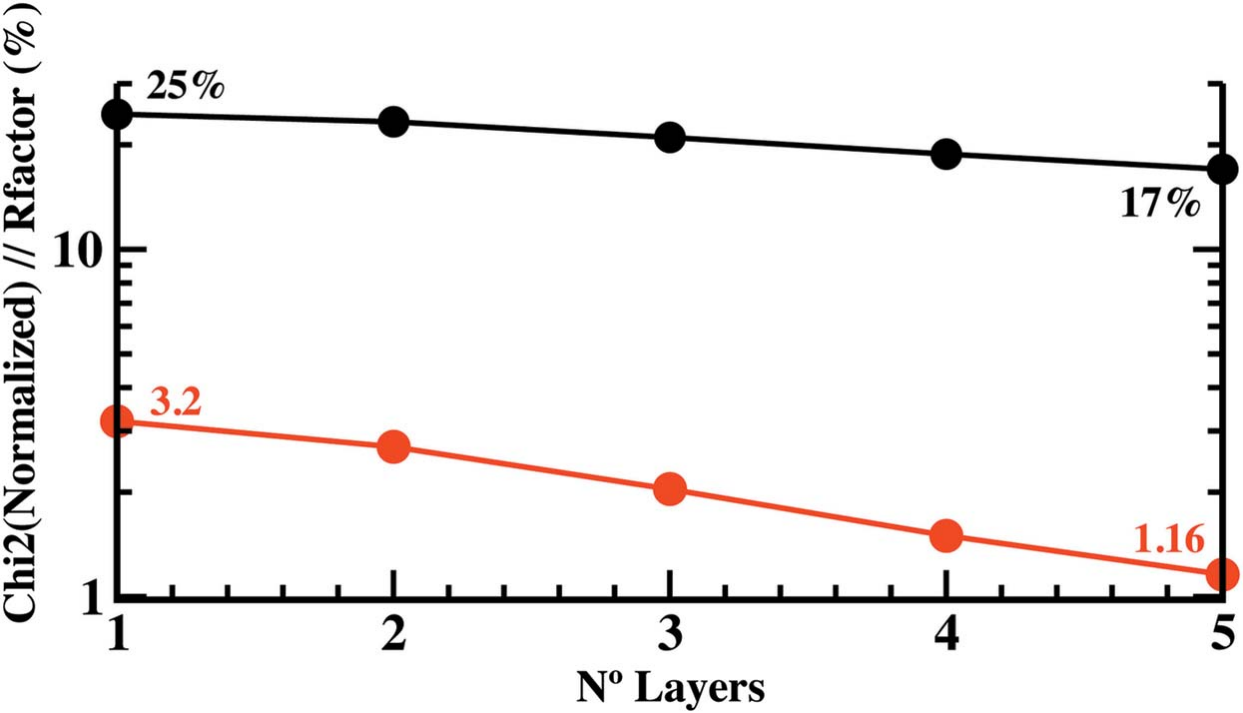
The evolution of 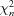 and *R* factor with the number of layers considered in the model.

The structure refinement of the pristine FAp_0001_ surface under dry conditions was initiated from an ideal FAp_0001_ termination. Relaxations were allowed layer by layer, gradually, starting from the surface and going into the bulk. The most favourable surface-layer termination was inspected by refining initially the experimental data set (CTRs) with each surface termination and their respective atomic occupancies (Table 1 ▸). Additional numbers of layers were considered, taking into account the progressive reduction with depth in the atomic distortions relative to their ideal positions. Fig. 1 ▸ shows the evolution of 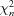 and *R* factor with the number of layers in the model. From the fifth layer, the 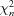 value does not improve appreciably.

**Figure 2:**
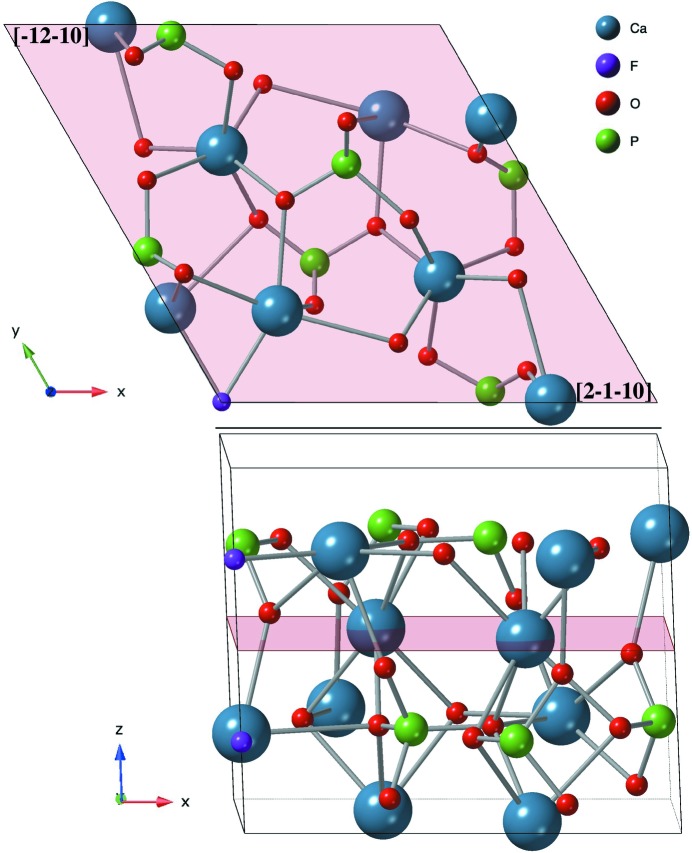
The FAp_0001_ bulk unit cell, viewed (top) from the top and (bottom) laterally. The cell shows the two block layers forming the cell. The groups of atoms belonging to each block are displayed separated by a plane.

The space group of FAp_0001_ is *P*6_3_/*m*. Both the *z* mirror plane (*m*) and the symmetry translation along the *z* direction, *i.e.* *z* + ½ (*P*6_3_ subgroup), were checked. However, the two space groups were dropped during the refinement because models constructed with *P*6_3_/*m* and *P*6_3_ symmetries could not adequately adjust the experimental data: their 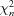 goodness-of-fit factors were twice that of our best 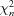. For this reason, the *P*3 symmetry of the 2D space group was selected to construct the final models used to adjust the data. The extra symmetry operations of *P*6_3_/*m* and *P*6_3_ [*i.e.* mirror plane or (*z* + ½) translation] reduce by almost a factor of two the number of (*x*, *y*, *z*) parameters with respect to the *P*3 symmetry: 36 parameters to define (*x*, *y*) coordinates (rotation angles and atomic shifts) plus 27 *z* parameters, a scale factor and a roughness factor (total = 65 parameters). Models considering the phosphate groups as rigid bodies can reduce the number of parameters by a factor of five (Torrelles *et al.*, 2004 ▸). Considering the *P*3 symmetry only, the structure of the surface retains a three-fold axis on the surface that provides different rotation values for atoms that are not located on the same level (the atoms related to the three-fold axis have identical *z* coordinates). With this symmetry a total of 107 structural parameters for the dry surface were used, *i.e.* 60 (*x*, *y*) parameters, 45 (*z*) parameters, a scale factor and a roughness parameter. The humid surface model has six (*x*, *y*, *z*) parameters more than the dry model, plus two occupancy factors to determine the position and surface coverage of the H_2_O and OH species present at the surface/interface. Although the number of parameters is large, we introduced bond-length restraints for [PO_4_] as a structurally invariant subunit with maximum tolerances up to ±0.15 Å. These restraints allow us to compensate for the lower sensitivity of some O atoms of the model in phosphate groups located in layers 2-*b*, 3-*a* and 4-*d* with respect to the others bonded to the same group (the definition of these labels is given in Table 2 ▸). The use of bond-length restraints facilitates the convergence of the fit towards a real structure while maintaining the P—O bond distances within a physical range with a maximum–minimum bond tolerance of 10% (Watkin, 1994 ▸). Fig. 2 ▸ shows the two blocks of atoms forming the bulk unit cell.

In previous work, the FAp–H_2_O interface has been probed by X-ray reflectivity (XRR) and CTR investigations (SXRD) with different sensitivities to the geometry of the surface structure (Pareek *et al.*, 2007 ▸; Park *et al.*, 2004 ▸). XRR measurements only yield a projection of the 2D structure onto 1D, with good information on layer distances but without information on the lateral structural periodicity. CTRs provide information on the mineral surface and on possible layers of adsorbates, provided these have the same lateral periodicity as the mineral surface. XRR studies of the FAp–H_2_O interface on a fully hydrated surface by Park *et al.* (2004 ▸) showed adsorption of two water layers at out-of-surface plane distances of 2.64 (9) and 4.17 (5) Å from the mineral surface. In our earlier SXRD study, specular and non-specular CTRs of the FAp(100) (FAp_100_)–H_2_O interface disclosed the presence of one laterally ordered water layer in a humid environment at an out-of-surface plane distance of 1.8 (1) Å (Pareek *et al.*, 2007 ▸), although specular X-ray reflectivity showed the presence of two water layers when fully hydrated (Park *et al.*, 2004 ▸), giving a 1D representation of the surface electronic density along the normal direction. In later work, the lateral ordering of these two water layers was demonstrated from a 3D analysis of the hydrated FAp_100_–water interface placed at distances from the relaxed surface of 1.6 (1) and 3.18 (10) Å (Pareek *et al.*, 2008 ▸). These refined surface models consider five surface blocks formed by ideal F—Ca—[PO_4_] {block = F(1)—Ca(5)—[PO_4_](3); the numbers in brackets indicate the number of elements of each type in a single block} as in the bulk. Each block contains two different Ca layers at different heights, and each bulk unit cell contains two of these blocks. Hence, each unit cell contains four layers with a spacing of one quarter of a unit cell (Δ*z* = 0.25) between them. See Fig. 2 ▸ for more details.

## 3. Results and discussion   

The starting model used to refine the data considers a surface slab with two and a half bulk unit cells. The initial positions of the atoms in the cell were the ideal bulk ones and the shifts from them follow *P*3 symmetry.

The surface termination under dry conditions was determined from a preliminary inspection of the evolution of 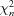 with each of the four possible termination layers (Table 1 ▸). The lowest values are obtained for F—Ca—[PO_4_] terminated block layers, while those terminating in two Ca atoms show higher χ^2^ values.

The best refined surface model includes five F—Ca—[PO_4_] surface blocks (or ten atomic layers). Under dry conditions, the topmost layer is Ca—PO_3_ terminated where no O and/or OH adsorption is observed to complete the typical octahedral coordination of the Ca cation (presence of a phosphite PO_3_ anion). The influence of this O atom during the refinement is also shown in Table 1 ▸ in terms of χ^2^.

The surface resembles an ideal bulk termination with relaxations in the top surface layers. The surface roughness value obtained with *ROD* and using the approximate β model (Vlieg, 2000 ▸; Robinson, 1986 ▸, 1998 ▸ and references therein) was small, implying that the FAp_0001_ surface is mostly flat (β = 0.04 ± 0.01). The tilts of the [PO_4_] tetrahedron were fixed to the *P*3 symmetry given by the model. In ideal (bulk terminated) FAp_0001_, the Ca polyhedron can be considered as a CaO_5_F octahedron, but, in addition, a seventh weak bond to O exists, redefining the polyhedron as CaO_5_F(O) (Hughes *et al.*, 1989 ▸). For the ideal truncated surface, this coordination reduces to four or five. Under dry conditions, the reduction in coordination causes a non-uniform charge distribution of the topmost Ca atoms, which introduces significant distortions in the atoms of the upper surface layers, as has already been observed in FAp_100_ (Pareek *et al.*, 2007 ▸).

The exposure of FAp_0001_ to an H_2_O saturated atmosphere results in a relaxed surface where the atoms are displaced inwards, *i.e.* along the *z* direction, perpendicular to the surface. The surface in a humid atmosphere is covered with a partially occupied H_2_O layer (three water molecules placed on top of each of the Ca atoms with an occupancy of 33%). In addition, three OH groups also appear on top of the P atoms with an occupancy of 33% each, thus completing the P coordination. The weight of the water and hydroxyl molecules in this surface was also tested in terms of χ^2^, as indicated in Table 1 ▸. Considering the symmetry of the apatite crystal structure and the morphology of naturally occurring apatites, the basal plane (100) is rarely developed, perhaps because of its comparatively fast growth kinetics. In order to keep the charge balanced and maintain the symmetry, a perfect cation or phosphate termination is not favoured and surface roughness is required to avoid charge separation. On the other hand, macroscopic adsorption experiments indicate that an equivalent of two monolayers of water are strongly adsorbed on microcrystalline apatite (Posner, 1985 ▸). The low water concentration detected at the (0001) surface is assigned to positional disorder, which could be the origin of this apparent small H_2_O/OH adsorption.

**Figure 3:**
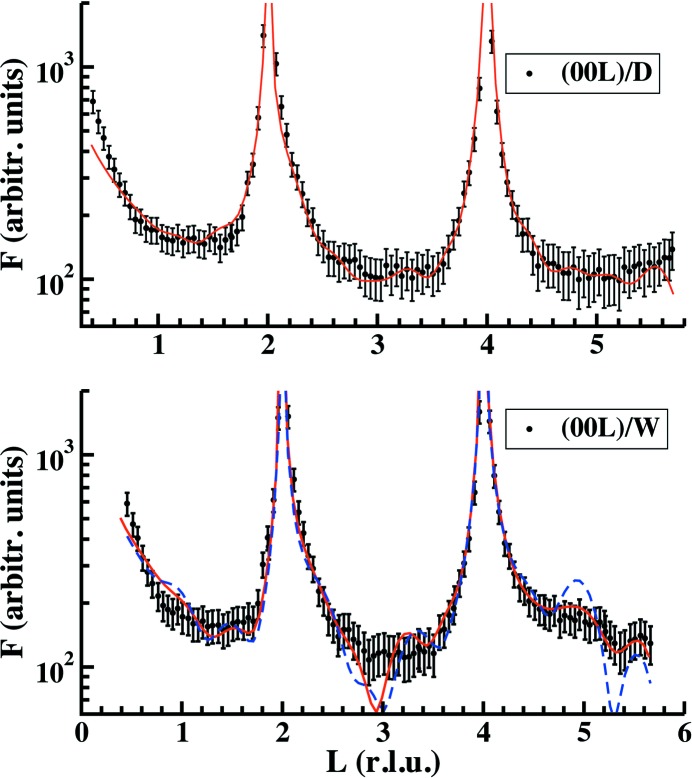
Reflectivity curves for the dry and humid cases. The curves have been refined together with the CTRs and a model considering two and a half bulk unit cells. The red and blue curves were obtained considering surface hydroxyl coverages of 100% or 33%, respectively. See text for more details.

To ensure both (i) the possible loss or not of one O atom bonded to the topmost phosphate unit in the dry surface and (ii) the percentage of the surface water and hydroxyl coverages (100% or 33%) on the hydrated surface, we have analysed the reflectivity curves measured for both cases. Both dry (top) and humid (bottom) curves are shown in Fig. 3 ▸. The dry reflectivity curve agrees with the results obtained from the analysis of the corresponding CTRs, confirming that one O atom bonded to the topmost phosphate unit is lost, thus forming a phosphite ion. The humid reflectivity curve indicates the presence of three water and three OH molecules per unit cell, well localized along the surface normal at 2.23 (1) Å above the topmost Ca layer. The red and blue curves in Fig. 3 ▸ (bottom) are obtained considering hydroxyl coverages of 100% or 33%, respectively. The red curve considering full coverage fits the experimental data slightly better. The corresponding CTRs measured for this case only detect one water and one OH molecule per unit cell, thus with an atomic occupancy of 0.33. As each molecule can be bonded to only one of the three Ca and P atoms forming part of the surface, the model places one molecule of each specimen with an occupancy factor of 1/3 on top of them. To reconcile this apparent discrepancy between CTRs and reflectivity, we performed an analysis of their corresponding temperature factors (Debye–Waller or DW, *B* = 8π^2^〈*u* ^2^〉). The model was then refined using both data sets and considering a similar anisotropic dependence of the DW for H_2_O and OH mol­ecules (in-plane and out-of-plane DW components *B*_par_ and *B*_perp_, respectively). The refinement process yields (*B*_par_, *B*_perp_) values of (15 ± 4 Å^2^, 1.2 ± 0.1 Å^2^) when considering occupancies of 1 for water and OH molecules. The value of *B* becomes isotropic (*B*_iso_ = 1.5 ± 0.1 Å^2^) when the occupancies of the molecules are 1/3. This analysis indicates that the H_2_O and OH molecules are well defined along the *z* direction but they show positional in-plane disorder.

**Figure 4:**
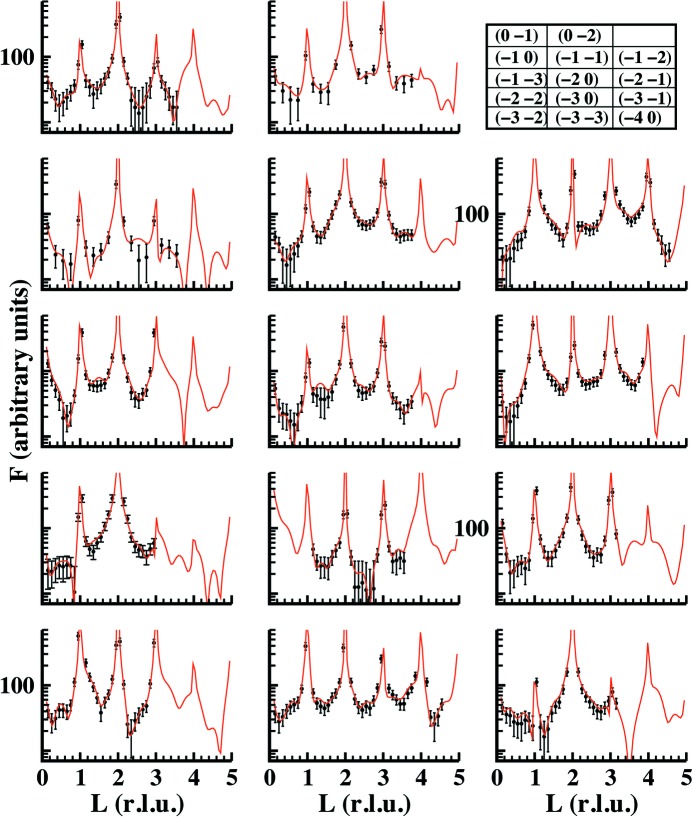
A comparison between experimental (black symbols with error bars) and calculated (red continuous lines) structure factors for FAp_0001_ under dry conditions. A standard deviation error of 13% was estimated from averaging between equivalent reflections. This value was also similar to that obtained for the humid case.

**Figure 5:**
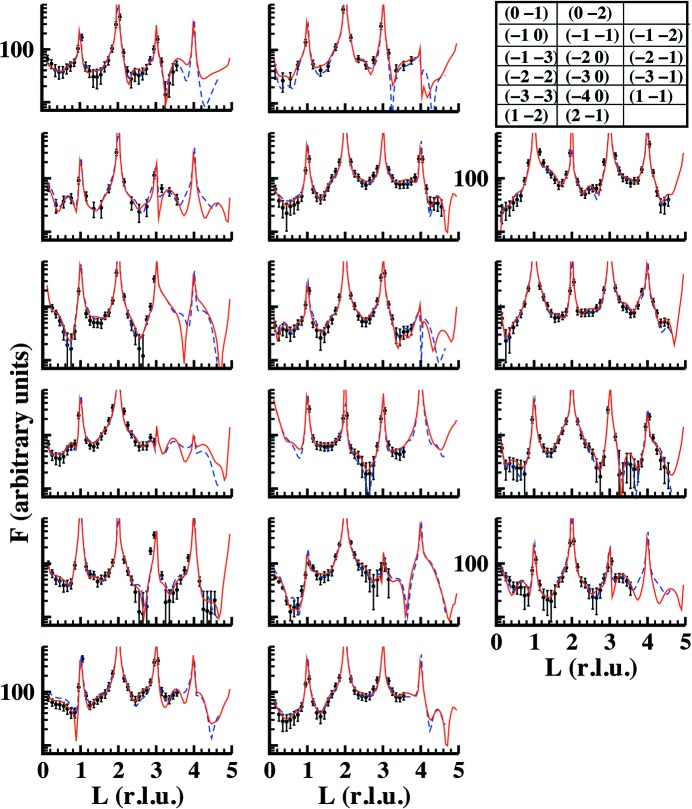
A comparison between experimental (black symbols with error bars) and calculated (red continuous lines) data for the humid FAp_0001_ surface using the water/humid model described in the main text.

**Figure 6:**
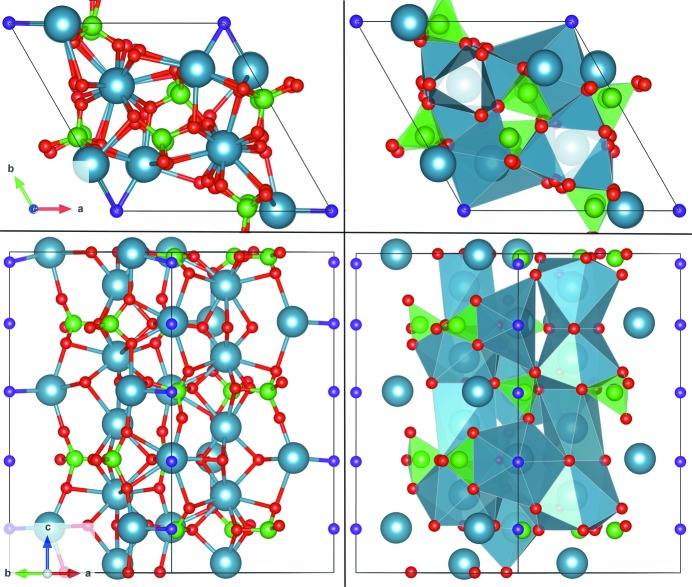
The surface structure of dry FAp_0001_. (Left) Ball-and-stick representations of (top) top and (bottom) lateral views. (Right) The same views in a polyhedral representation. Colours: P atoms are green, F violet, Ca blue and O red.

The refinements of the structure for the dry and humid data sets are shown in Figs. 4 ▸ and 5 ▸, respectively. The figures show the experimental and calculated structure factors for both cases using the full data sets formed by CTRs and reflectivity curves. Fig. 5 ▸ shows two curves obtained using two different models. Red curves consider water and hydroxyl molecules at the surface with occupancy 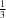 and *B*_iso_ = 1.5 Å^2^. In the second model (blue dashed curves) the occupancy factor of each H_2_O/OH molecule is 1 with (*B*_par_, *B*_perp_) values of (15 Å^2^, 1.2 Å^2^). As can be observed, the fit is slightly worse for reflections with high (*h*, *k*) values as a consequence of the absence of molecular disorder along the *z* direction, as detected from the analysis of the reflectivity measurements. From our best model (red curves), the Ca—H_2_O bond distances are about 2.28 (5) Å (Pareek *et al.*, 2007 ▸; Wolthers *et al.*, 2012 ▸; Kerisit *et al.*, 2003 ▸) and the OH—P bond lengths are 1.65 (5) Å (Gamoke *et al.*, 2009 ▸) thus completing the P coordination. The single P—OH bond is slightly longer than P=O (1.5 Å). The ideal atomic positions in fractional coordinates and the atomic displacements obtained after the refining process (using the *P*3 symmetry) of the FAp_0001_ surface structure under dry and humid conditions are shown in Table 2 ▸. Top and lateral views of the refined models are represented in Figs. 6 ▸ and 7 ▸. The evolution of 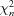 and *R*-factor figures of merit with the number of layers considered in the model during the refinement procedure (five F—Ca—[PO_4_] surface blocks) is shown in Fig. 1 ▸.

The atomic relaxations of the surface layers in Table 2 ▸ decrease when the depth increases. In the first two blocks, relaxation is dominated by the rotations of the phosphates around the *c* axis, as indicated in Table 3 ▸, which gives the rotation angles for each of the five [PO_4_] groups considered in our surface model for dry and water-saturated atmospheres. The average atomic shifts for the dry and water-saturated atmospheres relative to the ideal bulk positions are easily recognizable from the phosphorus tetrahedral rotation from the top (1) to the inner (5) surface layers (Table 3 ▸). The average evolution of the angles of the [PO_4_] units with depth shows that the water-saturated surface is less distorted than the dry one (Table 4 ▸). This behaviour is similar to that detected for FAp_100_ under dry and humid (RH ≃ 75%) conditions (Pareek *et al.*, 2007 ▸, 2008 ▸, 2009 ▸). From the analysis of the data it looks as if the distortion of the top [PO_4_] is relaxed as a function of surface depth to take up an almost regular tetrahedral shape again. Moreover, the dry surface shows oxygen-unsaturated phosphate units. The outermost surface P—O oxygen is lost, as determined from the structure refinement. The resulting [PO_3_]^3−^ ion unit (phosphite ion) shows average O—P—O bond angles close to 120°, as would be expected for this entity. The phosphite ion becomes a regular tetrahedron under a humid environment by partial adsorption of hydroxyl groups (one third of an OH group per PO_3_ unit = 33%). The experimental P—OH bond length is 1.65 Å, compatible with a value reported elsewhere (Gamoke *et al.*, 2009 ▸).

The atomic displacements reduce strongly for layers (4) and (5) in the same proportion as the rotation angles indicated in Table 3 ▸. The evolution of [PO_4_] rotation angles with depth on the humid surface shows a smoother evolution towards the bulk structure than that of the dry surface, as expected.

A summary of these distortions is presented in Table 4 ▸, which shows the evolution of the average bond angle and bond length with depth for the three topmost surface tetrahedral [PO_4_] units. As observed, the average angles of the distorted tetrahedra are close to the bulk ones, except for the topmost surface layer of the dry case, where the distortion is rather large due to the absence of one P—O bond leading to a [PO_3_] unit, with an average O—P—O angle of 112° (Table 4 ▸). On the humid surface, this situation is overcome by the partial adsorption of one hydroxyl molecule that stabilizes the charge distribution in the phosphate unit and reduces its distortion. The maximum/minimum bond-angle distortions for both cases are 123 and 94° (dry), and 121 and 100° (humid), which correspond to the topmost surface PO units.

Earlier X-ray diffraction studies of the FAp_100_–H_2_O interface on a fully hydrated surface show up to two layers of water molecules with (Park *et al.*, 2004 ▸) and without (Pareek *et al.*, 2007 ▸, 2008 ▸, 2009 ▸) lateral order. A direct comparison between the structural results obtained from these experiments and the results obtained in the present study cannot be made due to the different structural surface terminations: orthorhombic for FAp_100_ and hexagonal for FAp_0001_. In our work, we obtain a hexagonal dry surface free of O atoms on the topmost surface layer. The topmost [PO_4_] tetrahedron is in fact a [PO_3_] unit where the apical O atom is missing. Average O—P—O bond angles indicate that this [PO_3_] unit could be similar to the phosphite ion which has an average bond angle of ∼112°. Moreover, the hydrated surface does not show a fully ordered layer of H_2_O or OH molecules on top of Ca and P, respectively, but rather partial occupancies. The adsorbed molecules show a rather large thermal vibration amplitude, as discussed previously, whose impact on the data is the attenuation of the intensity profiles of the CTRs when their atomic weight increases. This intensity attenuation smoothes the profile of the CTRs, reducing the amplitude of the oscillations or features coming from the overlay. Since the atomic weight of H_2_O/OH molecules is coupled with their corresponding DW values, the quantification of the water surface layer is difficult, which is equivalent to supposing a disordered presence of the topmost water or hydroxyl molecules around the Ca and P specimens.

In this case, the distance of the H_2_O monolayer from the relaxed surface is 2.23 Å, which is larger than that obtained by Pareek *et al.* (2007 ▸) (1.8 Å) but much shorter than that obtained by Park *et al.* (2004 ▸) (2.64 Å) from reflectivity data. The presence of a second H_2_O layer was not detected, probably due to the presence of large areas of the surface where the molecules are mainly disordered. The effective ratio of disordered H_2_O:OH molecules per unit cell is 1:1. Comparing the differential heats of adsorption of water for FAp/HAp(0001) and pristine (010) surfaces they are very similar (Posner, 1985 ▸; Corno *et al.*, 2009 ▸). For both surfaces, the heat of vaporization for water (close to 10 kcal mol^−1^; 1 kcal mol^−1^ = 4.184 kJ mol^−1^) is reached when two water molecules per unit cell are adsorbed. For our experimental FAp(0001) case, we only detect a 33% water occupancy per Ca atom at the surface (and 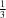 OH molecule per [PO_3_] unit at the surface). Most likely, the apparent deficiency of water molecules reflected by the CTR data set on our FAp(0001) surface is due to positional disorder. The FAp(100) cell definition (Pareek *et al.*, 2007 ▸), identical to the pristine (010) surface (Corno *et al.*, 2009 ▸), also shows two ordered water molecule layers at the surface, suggesting a value of heat of vaporization for water similar to those of pristine (010) and FAp/HAp(0001) surfaces.

## 4. Conclusions   

The surface/interface structure of FAp_0001_ under dry (He environment) and humid (RH ≃ 100%) conditions has been probed with SXRD. The surface model built to simulate the dry and water exposed surfaces is formed by a slab of five blocks with the chemical formula Ca_5_(PO_4_)_3_F that forms a layered structure along the surface normal direction with a length of 2.5 times the *c*-axis value for Fap(0001). Under dry conditions, our findings indicate that the surface resembles an ideal bulk termination with relaxations in the top surface layers. This surface is compatible with a [PO_3_] phosphite ion termination where the P atom is neutral (one orbital is occupied by two electrons and each of the other three is bonded to one O atom that is negatively charged). When the surface is exposed to wet conditions, the analysis of reflections with *h* or *k* values differing from zero shows Ca and P atoms bonded to a partially occupied water or hydroxyl OH layer (33%), respectively. Analysis of the reflectivity data for the humid case shows a surface fully covered with water and hydroxyl molecules: three bonded to the Ca atoms (2.28 Å) and the other three to the P atoms (1.65 Å), respectively. Reflectivity data show that the mol­ecules are placed at well defined heights from the topmost surface layer with a small DW value (*B*_perp_ = 1.2 Å^2^). CTRs show similar positions for both molecules but with lower occupancies that we interpret as in-plane positional disorder. This positional disorder is compatible with a high molecular in-plane DW value (*B*_par_ = 15 Å^2^) or a reduction of their respective occupancy weight by a factor of three.

In wet conditions the reaction of the hydroxyl OH ion with the phosphite [PO_3_] ion produces a phosphate [PO_4_] ion termination. The sensitivity of the data to other water molecules adsorbed on the wet surface, *i.e.* a second layer of water molecules, would be smaller than those already adsorbed on the topmost surface layer as a consequence of the high disorder already existing in this layer.

The significant difference between the structures of the FAp_100_ and FAp_0001_ surfaces is the explicit presence of dis­ordered adsorbed water and hydroxyl molecules on the outermost {0001} surface layer compared with the {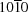} surface of mineral specimens from the same locality. Also, the (0001) surface is very rare, whereas the (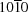) prism face is a common feature of the apatite morphology. The ratio of surface area reflects the growth rate and is also related to the surface energy. Based on our results, the (0001) face with its incomplete hydration obviously favours further growth. In addition, fast growth of the {0001} face of apatite leads to a pronounced development of the pyramidal faces {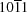}.

Our results are also in agreement with the observation that bone apatite fibres grow preferentially along [0001] while the {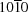} faces are inhibited, because the perfect surface interacts with collagen or non-collagenous protein inhibiting further growth, giving a means of controlling the size and morphology of apatite nanocrystals in bone (Xie & Nancollas, 2010 ▸). In an experimental study of glycine/H_2_O at face (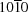) using grazing-incidence XRD structure analysis, charge matching of the amino acid with the Ca and phosphate ions has been shown to form a periodically ordered layer shielding the surface from the nutrient solution (Pareek *et al.*, 2009 ▸). Obviously, the molecular disorder of light molecules such as H_2_O and OH detected on the (0001)-surface is higher than that induced on the (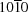) surface by heavier organic molecules. This lower ordering could be consequence of their higher surface mobility under flowing vapor conditions as experimentally detected in the form of an anomalous increase of their in-plane Debye Waller.

## Supplementary Material

Crystal structure: contains datablock(s) FAP0001_dry, FAP0001_humid. DOI: 10.1107/S2052520619010412/dq5039sup1.cif

Structure factors: contains datablock(s) FAP0001_dry. DOI: 10.1107/S2052520619010412/dq5039FAP0001_drysup2.hkl

Structure factors: contains datablock(s) FAP0001_humid. DOI: 10.1107/S2052520619010412/dq5039FAP0001_humidsup3.hkl

CCDC references: 1942347, 1942348

## Figures and Tables

**Figure 7:**
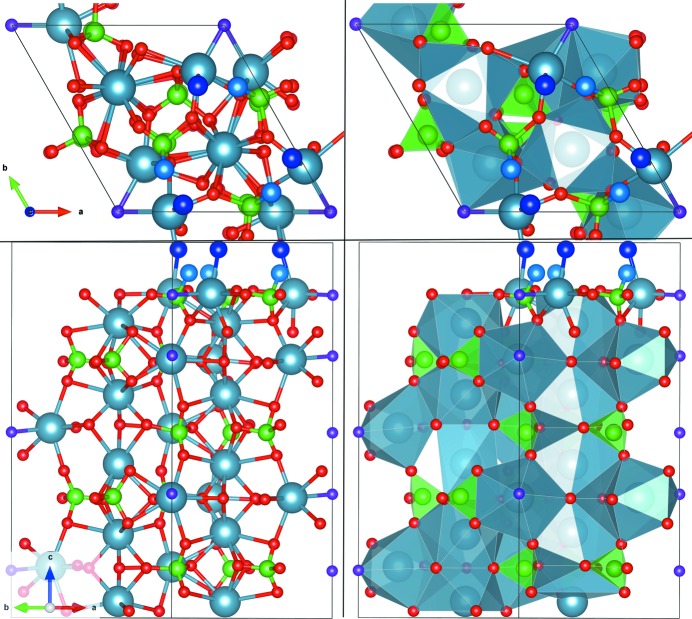
The surface structure of humid FAp_0001_. (Left) Ball-and-stick representations of (top) top and (bottom) lateral views. (Right) The same views in a polyhedral representation. Colours: P atoms are green, F violet, Ca blue, O red, H_2_O dark blue and OH light blue.

**Table:** 

Dry	1st layer, O = 0	1st layer, O = 1	2nd layer	3rd/4th layer
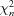	1.2	2.2	6.1	2.5/2.8

**Table:** 

Humid	H_2_O = 0, OH = 0	H_2_O = 0, OH = 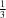	H_2_O = 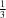 , OH = 0	H_2_O = 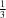 , OH = 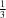
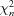	1.5	1.4	1.3	1.16

**Table 2:** Atomic coordinates of the FAp_0001_ surface slab formed by two and a half bulk cells The columns on the left show the ideal positions of the surface atoms in the bulk, (*x*, *y*, *z*)_bulk_. After refining the experimental data sets with dry and humid surfaces with their respective models, the atomic shifts with respect to the ideal positions are given as (Δ*x*, Δ*y*, Δ*z*)_dry_ and (Δ*x*, Δ*y*, Δ*z*)_humid_, respectively. The error bars assigned to each shift are given by the corresponding subscript index (Torrelles *et al.*, 2004 ▸). Atomic positions and shifts are given in crystallographic coordinates with respect to the hexagonal cell defined by the lattice parameters (9.375 Å, 9.375 Å, 6.887 Å, 90°, 90°, 120°). By adding or subtracting (according to the sign indicated in the table) the corresponding shifts to/from the ideal atomic positions, the position of the fitted atom in the unit cell is obtained. Dashes (–) denote that the atom does not exist in the model, and asterisks (*) denote that the corresponding coordinate was kept fixed during the refinement procedure due to symmetry restrictions (atoms placed on a ternary axis). Atomic coordinates marked with an asterisk also indicate that the corresponding atom was placed on a ternary axis. Ca_*i*-*j*_ denotes Ca atom *j* in a layer *i*. P_*i*-(*a*→*e*)_ denotes a P atom in a layer *i*. The additional labels *a* to *e* help to identify the four O atoms bonded tetrahedrally to each P atom: O_*a*-t_ (oxygen *a*, top), O_*a*-1_ and O_*a*-2_ are oxygens placed on the same *z* level as the P atom and O_*a*-b_ (oxygen *a*, bottom).

	Surface atomic coordinates								
Atom label	*x*_bulk_	*y*_bulk_	*z*_bulk_	Δ*x*_dry_	Δ*y*_dry_	Δ*z*_dry_	Δ*x*_humid_	Δ*y*_humid_	Δ*z*_humid_
O*	0.2419	0.9929	3.5292	–	–	–	0.05_2_	0.03_2_	0.03_1_
F	0.0000	0.0000	3.2500	*	*	−0.030_6_	*	*	−0.030_6_
Ca_1_	0.2419	0.9929	3.2500	−0.007_2_	−0.006_2_	−0.004_2_	0.002_2_	0.007_2_	−0.018_2_
P_1-*a*_	0.3985	0.3690	3.2500	0.005_5_	0.011_5_	−0.008_3_	−0.004_3_	−0.011_2_	−0.035_4_
O_*a*-t_	0.3411	0.2571	3.4292	–	–	–	−0.03_2_	−0.04_2_	−0.05_1_
O_*a*-1_	0.3269	0.4844	3.2500	0.007_5_	0.018_5_	0.004_5_	0.004_6_	−0.026_5_	−0.026_4_
O_*a*-2_	0.5876	0.4655	3.2500	−0.011_5_	−0.032_5_	−0.025_4_	0.002_5_	0.027_4_	−0.007_5_
O_*a*-b_	0.3411	0.2571	3.0708	0.018_5_	0.016_4_	0.017_3_	0.030_5_	0.002_4_	−0.026_4_
Ca_2-1_	0.3333	0.6667	3.0011	*	*	0.027_3_	*	*	0.014_3_
Ca_2-2_	0.6667	0.3333	2.9989	*	*	−0.003_3_	*	*	0.019_3_
F	0.0000	0.0000	2.750	*	*	−0.005_7_	*	*	0.025_6_
Ca_3_	0.7581	0.0071	2.750	−0.011_2_	−0.002_2_	0.009_2_	−0.2	−0.009_2_	0.010_2_
P_2-*b*_	0.6016	0.6310	2.750	0.009_3_	−0.007_3_	−0.007_3_	−0.015_3_	−0.002_3_	0.007_4_
O_*b*-t_	0.6589	0.7429	2.9292	0.014_5_	−0.008_5_	−0.037_4_	0.006_6_	0.017_5_	0.015_4_
O_*b*-1_	0.6731	0.4844	2.7500	−0.013_5_	−0.014_5_	−0.003_4_	0.018_6_	0.037_5_	−0.013_4_
O_*b*-2_	0.4124	0.5345	2.7500	0.020_5_	0.015_5_	−0.003_4_	−0.013_5_	−0.021_4_	−0.028_5_
O_*b*-b_	0.6589	0.7429	2.5708	−0.013_5_	−0.011_5_	0.028_3_	−0.008_5_	0.006_4_	0.024_4_
Ca_4-1_	0.6667	0.3333	2.5011	*	*	0.016_2_	*	*	0.005_2_
Ca_4-2_	0.3333	0.6667	2.4989	*	*	0.010_2_	*	*	0.001_2_
F	0.0000	0.0000	2.2500	*	*	0.006_7_	*	*	−0.027_7_
Ca_5_	0.2419	0.9929	2.2500	0.006_2_	−0.001_2_	−0.000_2_	0.004_2_	0.007_2_	0.006_2_
P_3-*c*_	0.3985	0.3690	2.2500	0.006_3_	−0.001_2_	−0.012_3_	0.016_3_	0.001_2_	−0.017_3_
O_*c*-t_	0.3411	0.2571	2.4292	0.014_5_	−0.003_4_	−0.020_3_	0.017_5_	0.039_4_	−0.008_4_
O_*c*-1_	0.3269	0.4844	2.2500	−0.006_5_	−0.013_5_	0.031_3_	0.005_5_	0.003_5_	0.033_4_
O_*c*-2_	0.5876	0.4655	2.2500	0.016_5_	0.007_5_	0.000_5_	0.000_5_	0.001_4_	0.012_5_
O_*c*-b_	0.3411	0.2571	2.0708	−0.005_5_	0.007_5_	0.004_4_	0.020_6_	0.021_5_	0.000_4_
Ca_6-1_	0.3333	0.6667	2.0011	*	*	0.010_3_	*	*	0.003_3_
Ca_6-2_	0.6667	0.3333	1.9989	*	*	−0.008_3_	*	*	−0.006_3_
F	0.0000	0.0000	1.7500	*	*	−0.004_7_	*	*	0.030_6_
Ca_7_	0.7581	0.0071	1.7500	−0.003_3_	−0.004_3_	0.008_1_	−0.006_2_	−0.006_2_	0.007_2_
P_4-*d*_	0.6016	0.6310	1.7500	−0.004_3_	0.001_2_	−0.003_3_	−0.005_2_	0.001_2_	0.015_2_
O_*d*-t_	0.6589	0.7429	1.9292	0.002_5_	−0.020_5_	−0.017_4_	0.009_6_	0.003_5_	0.014_5_
O_*d*-1_	0.6731	0.4844	1.7500	0.003_5_	0.008_5_	−0.012_4_	0.016_5_	0.037_4_	−0.010_4_
O_*d*-2_	0.4124	0.5345	1.7500	−0.009_5_	−0.006_5_	0.003_4_	0.008_5_	−0.008_4_	−0.015_4_
O_*d*-b_	0.6589	0.7429	1.5708	−0.014_5_	−0.014_4_	0.003_3_	−0.003_5_	−0.005_4_	−0.001_4_
Ca_8-1_	0.6667	0.3333	1.5011	*	*	−0.007_2_	*	*	0.005_2_
Ca_8-2_	0.3333	0.6667	1.4989	*	*	−0.003_2_	*	*	−0.012_2_
F	0.0000	0.0000	1.2500	*	*	0.008_6_	*	*	−0.027_6_
Ca_9_	0.2419	0.9929	1.2500	−0.002_2_	−0.003_2_	0.002_1_	0.006_2_	0.007_2_	0.005_2_
P_5-*e*_	0.3985	0.3690	1.2500	0.004_3_	0.001_2_	−0.007_3_	0.005_2_	0.002_2_	−0.004_2_
O_*e*-t_	0.3411	0.2571	1.4292	0.014_4_	−0.006_4_	−0.015_3_	0.013_4_	0.019_4_	−0.010_4_
O_*e*-1_	0.3269	0.4844	1.2500	0.011_5_	−0.003_5_	0.015_4_	0.005_5_	0.003_4_	−0.005_4_
O_*e*-2_	0.5876	0.4655	1.2500	0.011_4_	0.008_5_	−0.006_4_	−0.009_4_	−0.003_4_	0.013_4_
O_*e*-b_	0.3411	0.2571	1.0708	0.010_5_	0.009_4_	0.001_3_	0.014_5_	0.017_4_	0.009_4_
Ca_10-1_	0.3333	0.6667	1.0011	*	*	0.000_2_	*	*	0.006_2_
Ca_10-2_	0.6667	0.3333	0.9989	*	*	0.006_2_	*	*	−0.001_2_
O	0.6589	0.7429	0.9294	0.002_4_	−0.010_4_	−0.009_3_	−0.001_5_	−0.005_4_	−0.003_4_

**Table 3:** Average rotation angles for each of the PO_4_/PO_3_ tetrahedral/phosphite (dry case) units at the surface versus depth for dry and humid cases The signs + and − indicate the anticlockwise or clockwise rotation direction, respectively. The labels indicate the positions of the phosphate groups in the cell according to Table 2 ▸. The asterisk denotes the presence of a PO_3_^−^ ion as obtained from the analysis. Error bars are obtained from a 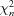 analysis (Feidenhans’l, 1989 ▸).

PO_4_	(PO_4_)_1-*a*_/(PO_3_)_1-*a*_	(PO_4_)_2-*b*_	(PO_4_)_3-*c*_	(PO_4_)_4-*d*_	(PO_4_)_5-*e*_
Dry (± 0.8°)	(+) 1.1*	(+) 1.1	(−) 1.3	(−) 1.8	(−) 0.3
Humid (± 0.8°)	(+) 1.4	(−) 1.1	(−) 1.7	(−) 0.8	(−) 0.5

**Table 4:** Evolution of the average bond angles (°) and bond lengths (Å) for the three topmost surface phosphate units obtained from the atomic coordinates given in Table 2 ▸ The phosphate unit of the topmost surface layer (PO_3_)_1-*a*_ of the dry surface is formed by three P—O bonds (marked with an asterisk). The angle is close to that expected for the phosphite ion, PO_3_^3−^ (120°). For the humid case, the hydroxyl molecule has been considered to compute the average bond angle and bond length. The average bond angles and bond lengths of the bulk phosphate PO_4_ unit are 109.5° and 1.50 Å, respectively. Bond-angle and bond-length errors were calculated from Carpenter (1979 ▸).

PO_4_	(PO_4_)_1-*a*_/(PO_3_)_1-*a*_	(PO_4_)_2-*b*_	(PO_4_)_3-*c*_
Dry; angle, length (± 0.5°, ± 0.08 Å)	112.2, 1.48*	109.1, 1.46	109.1, 1.52
Humid; angle, length (± 0.5°: ± 0.08 Å)	109.5, 1.50	109.1, 1.60	108.7, 1.46

## References

[bb1] Ben-Nissan, B. (2014). Editor. *Advances in Calcium Phosphate Biomaterials*. Heidelberg: Springer.

[bb2] Bragg, L., Claringbull, G. F. & Taylor, W. H. (1965). *The Crystalline State*, Vol. IV, *Crystal Structures of Minerals*. Cornell University Press.

[bb3] Calderín, L., Stott, M. J. & Rubio, A. (2003). *Phys. Rev. B*, **67**, 134106.

[bb4] Carpenter, G. B. (1979). *Acta Cryst.* A**35**, 248–250.

[bb5] Combes, C., Cazalbou, S. & Rey, C. (2016). *Minerals*, **6**, 34.

[bb6] Corno, M., Busco, C., Bolis, V., Tosoni, S. & Ugliengo, P. (2009). *Langmuir*, **25**, 2188–2198. 10.1021/la803253k 19161264

[bb7] Elliot, J. C. (1994). *Structure and Chemistry of the Apatites and Other Calcium Orthophosphates*. Amsterdam: Elsevier Science.

[bb8] Feidenhans’l, R. (1989). *Surf. Sci. Rep.* **10**, 105–188.

[bb9] Gamoke, B., Neff, D. & Simons, J. (2009). *J. Phys. Chem. A*, **113**, 5677–5684. 10.1021/jp810014s 19378976

[bb27] Hahn, Th. (1996). Editor. *International Tables for Crystallography*, Vol. A, *Space-group symmetry*, 4th ed. Dordrecht: Kluwer.

[bb10] Haldar, S. K. & Tišljar, J. (2014). *Introduction to Mineralogy and Petrology*, ch. 2, *Basic Mineralogy*. Amsterdam: Elsevier.

[bb12] Hughes, J. M., Cameron, M. & Crowley, K. D. (1989). *Am. Mineral.* **74**, 870–876.

[bb13] Kerisit, S., Parker, S. C. & Harding, H. C. (2003). *J. Phys. Chem. B*, **107**, 7676–7682.

[bb14] Lübke, A., Enax, J., Loza, K., Prymak, O., Gaengler, P., Fabritius, H.-O., Raabe, D. & Epple, M. (2015). *RSC Adv.* **5**, 61612–61622.

[bb15] Lübke, A., Loza, K., Patnaik, R., Enax, J., Raabe, D., Prymak, O., Fabritius, H.-O., Gaengler, P. & Epple, M. (2017). *RSC Adv.* **7**, 6215–6222.

[bb17] Pareek, A., Torrelles, X., Angermund, K., Rius, J., Magdans, U. & Gies, H. (2008). *Langmuir*, **24**, 2459–2464. 10.1021/la701929p 18278952

[bb16] Pareek, A., Torrelles, X., Angermund, K., Rius, J., Magdans, U. & Gies, H. (2009). *Langmuir*, **25**, 1453–1458. 10.1021/la802706y 19118469

[bb18] Pareek, A., Torrelles, X., Rius, J., Magdans, U. & Gies, H. (2007). *Phys. Rev. B*, **75**, 035418.

[bb19] Park, C., Fenter, P., Zhang, Z., Cheng, L. & Sturchio, N. C. (2004). *Am. Mineral.* **89**, 1647–1654.

[bb20] Posner, A. S. (1985). *J. Biomed. Mater. Res.* **19**, 241–250. 10.1002/jbm.820190307 3001090

[bb22] Renzi, M. de, Manzanares, E., Marin-Monfort, M. D. & Botella, H. (2016). *RSC Adv.* **6**, 74384–74388.

[bb23] Robinson, I. K. (1986). *Phys. Rev. B*, **33**, 3830–3836. 10.1103/physrevb.33.3830 9938797

[bb24] Robinson, I. K. (1998). *Acta Cryst.* A**54**, 772–778.

[bb25] Ruttenberg, K. C. & Berner, R. A. (1993). *Geochim. Cosmochim. Acta*, **57**, 991–1007.

[bb26] Stout, G. H. & Jensen, L. H. (1968). *X-ray Structure Determination*. New York: McMillan.

[bb28] Torrelles, X., Barrena, E., Munuera, C., Rius, J., Ferrer, S. & Ocal, C. (2004). *Langmuir*, **20**, 9396–9402. 10.1021/la048979f 15461535

[bb29] Vlieg, E. (2000). *J. Appl. Cryst.* **33**, 401–405.

[bb30] Watkin, D. (1994). *Acta Cryst.* A**50**, 411–437.

[bb31] Wolthers, M., Di Tommaso, D., Du, Z. & de Leeuw, N. H. (2012). *Phys. Chem. Chem. Phys.* **14**, 15145–15157. 10.1039/c2cp42290e 23042085

[bb32] Xie, B. & Nancollas, G. H. (2010). *Proc. Natl Acad. Sci. USA*, **107**, 22369–22370. 10.1073/pnas.1017493108 PMC3012503 21169505

